# Role of vitamin D on cancer immunotherapy

**DOI:** 10.3389/fendo.2012.00132

**Published:** 2012-11-16

**Authors:** Ashraf Karbasi, Amin Saburi

**Affiliations:** ^1^Baqiyatallah Research Center of Gastroenterology and Liver Disease, Baqiyatallah University of Medical SciencesTehran, Iran; ^2^Chemical Injuries Research Center, Baqiyatallah University of Medical SciencesTehran, Iran

Dear Editor in Chief,

With a great interest, we read a recently published paper in Frontier in Endocrinology journal by Vuolo et al., entitled “Vitamin D and Cancer” (Vuolo et al., 2012). They skillfully reviewed the role of vitamin D in all aspects of cancer such as cancer prevention, immune system and cancer, reverse effect of cancer on vitamin D, and vitamin D status in some important type of malignancy. This issue is a hot topic in oncology, but in this study there are some concerns which undermine the results to make a definite conclusion.

Although they concluded that “The immune system seems to represent a relevant target for the antineoplastic effects of vitamin D,” (Vuolo et al., 2012) they did not consider anti-neoplastic role of activated form of vitamin D using for immunotherapy of cancer. It was recently shown that a group of vitamin D-binding protein (DBP) so called Gc protein is an important precursor for activating macrophage as one of the main innate immune cell for cancer immunotherapy (Nagasawa et al., 2005). Although the aim of the review by Vuolo et al. was the role of Vitamin D in cancer, it was confirmed that the level of vitamin D is related to Vitamin D binding proteins (Carpenter et al., 2012).

In normal tissue, when a neoplastic tissue is arise, DBP is naturally activated by glycosylation and transform to DBP-derived macrophage-activating factor (Gc-MAF) by b-galactosidase and sialidase of B and T cells, respectively, which can activate macrophage to phagocyte the neoplastic cells. Group-specific component protein (Gc) as DBP preserve vitamin D in body fluids and put it available for tissues. But an enzyme (a-N-acetylgalactosaminidase) produced by neoplastic cells deactivate and denaturize this factor and provide neoplastic cells to spread (Yamamoto and Naraparaju, 1998; Ghanei et al., 2012). In some clinical studies, Gc-MAF has successfully been used for prostate, colorectal, and breast cancer (Yamamoto and Suyama, 2008; Yamamoto et al., 2008a,b). Therefore, vitamin D has a prominent role in natural tumorocidal activity of immune system as an initial precursor.
